# Traditional Khmer Medicine and its role in wildlife use in modern-day Cambodia

**DOI:** 10.1186/s13002-022-00553-5

**Published:** 2022-09-24

**Authors:** Thona Lim, Elizabeth Oneita Davis, Brian Crudge, Vichet Roth, Jenny Anne Glikman

**Affiliations:** 1 Free the Bears, Phnom Penh, Cambodia; 2 San Diego Zoo Wildlife Alliance, San Diego, CA USA; 3 Department of Natural Sciences and Environmental Health, University of South-Eastern Norway, Bø, Norway; 4 Instituto de Estudios Sociales Avanzados (IESA-CSIC), Córdoba, Spain

**Keywords:** Animism, *Boramey*, Consumer demand, *Kru Khmer*, Maternal healthcare, Spiritual healing, Sustainable use, Wildlife trade

## Abstract

Individuals across Cambodia depend on the use of natural products in Traditional Khmer Medicine (TKM), a traditional medicine system in Cambodia that has been practiced for hundreds of years. Cambodia is rich in fauna and flora species, many of which have been, and continue to be, traded domestically for traditional medicine use. Combined with other known exploitative practices, such as snaring for wild meat consumption and international trade in wildlife, domestic trade in wildlife medicine threatens populations of regional conservation importance. Here, we provide an updated understanding about how TKM is practiced in modern times; how TKM practices are transmitted and adapted; and roles of wildlife part remedies in TKM historically and presently. We conducted semi-structured interviews with TKM practitioners in Stung Treng, Mondulkiri Province, and at the National Center for Traditional Medicine in Phnom Penh, the capital of Cambodia. TKM is generally practiced in the private sector and is mostly informal, without enrollment in any academic training. TKM practitioner roles commonly involve collecting, preparing, selling, and advising on medicine, rather than providing direct treatment. Over half of the interviewed TKM practitioners (57.6%) were still prescribing wildlife parts as medicine over the past 5 years, with 28 species of wild animals reported. Lorises and porcupine were the wildlife products cited as being in highest demand in TKM, primarily prescribed for women’s illnesses such as post-partum fatigue (*Toas and Sawsaye kchey*). However, the supply of wildlife products sourced from the wild was reported to have dropped in the 5 years prior to the survey, which represents an opportunity to reduce prescription of threatened wildlife. We suggest that our results be used to inform tailored demand reduction interventions designed to encourage greater reliance on biomedicine and non-threatened plants, particularly in rural areas where use of biomedicine may still be limited.

**Supplementary Information:**

The online version contains supplementary material available at 10.1186/s13002-022-00553-5.

## Introduction

Natural resources play important and extensive roles in human daily life and are used in many different ways such as for food, entertainment, clothing, and medicine [1, 2]. Traditional Medicine (TM) refers to medicinal systems that rely heavily upon gathered plants and/or animals [1]. Worldwide, TM has been used to treat illnesses for thousands of years [3, 4]. TM continues to play a significant role throughout the world, with millions of individuals relying on TM as their primary healthcare option, especially in rural areas where natural resources are part of lives and culture [5, 6].

Cambodia, situated in mainland Southeast Asia, is classed as a Least Developed Country and is largely ethnically and religiously homogenous, with most of the population identifying as Khmer (96%) and Buddhist (97%) [7]. The Khmer are believed to have been one of the original peoples to settle in Southeast Asia, and consequently they have a long history within the region [8]. Traditional Khmer Medicine (TKM) was formed during the Angkor period (nine–fifteenth century AD) by directly incorporating components of Ayurvedic Medicine from India and Traditional Chinese Medicine (TCM), and combining with local beliefs and superstitions of the ancient Khmer medical system, which was informed by ancient Khmer animism [9, 10]. Animism, or belief in spirits within the natural and supernatural worlds, is a general term for ethnically and culturally specific religious belief systems across the world, where souls and/or spirits are attributed to aspects of the natural world, such as the forest (e.g. [11]). This basic definition of animism encompasses belief systems practiced to date across Southeast Asia, including within Cambodia’s neighbouring countries of Laos and Vietnam [12].

In rural areas of Cambodia, people still depend on this traditional medical system for their healthcare, with an estimated 40–50% of the Cambodian population using TM [13]; however, nowadays, individuals are less likely to actively consult TM practitioner and will instead self-prescribe herbal medicine treatments [14]. Traditional Khmer Medicine (TKM) comprises four primary forms of care that include: providing medicinal bases, dermabrasive practices, maintenance of hot/cold (“yin/yang”) balance, and supernaturalistic treatments such as spirit offerings [15, 16]. Supernaturalistic treatments are prescribed by traditional healers (known in Khmer language as “*Kru Khmer*”) or by Buddhist monks to treat the illnesses, which are believed to be caused by ghosts or spirits and are commonly related to Buddhist practices [17].

The ancient TKM medical texts were written in the Pali language, on palm-leaf that could be found at the pagoda libraries; however, these texts were mostly destroyed by the wars in Cambodia [18]. The ancient TKM of the Angkor era was changed during French colonization of Cambodia in the mid-1800s during which Western medicine was introduced to the country, although the local population resisted the colonizer’s medicine and maintained practicing TKM [19, 20]. Subsequently, during one of the most significant events in Cambodia’s recent history—the Khmer Rouge regime and Cambodian genocide—between 1975 and 1979, pre-existing French-style Western medicine was almost destroyed, with doctors or nurses forced to move to the countryside to work on agriculture or be killed. The Khmer Rouge encouraged a new system of healthcare that was neither fully TKM nor Western medicine, called “Khmer Rouge medicine” or “Revolutionary medicine” [19–21]. Khmer Rouge allowed the Indigenous healer—*Kru Khmer* to practice healthcare—under a bureaucratic structure, with limited services (performing the spiritual practices and providing family healthcare) and TM was manufactured into pills (also called “rabbit dropping” medicine) [19, 20]. Following the dissolution of Khmer Rouge, the influx of NGOs into Cambodia in the 1990s "fast-tracked" the healthcare system, leading to variation in the healthcare available and, it is argued, plurality in which services are utilized, valued, and respected [14]. Against this backdrop, the underlying medical worldwide in Cambodia, whether Western medicine or TM (or both) are being employed, is that illness is due to “social and moral transgressions” and that healing is an "active" process between “healer and sufferer” [19]. It has been further argued that every Khmer, even those who are higher status and may "scoff" at TM, uses Western medicine according to a traditional conceptualisation, e.g. where having a variety of treatments of varying mixtures is considered most effective at curing one’s health [19]; thus, medical pluralism, where both Western medicine and TM are used concurrently, is not uncommon [22].

According to [6], more than 1500 animal species are used in TCM, while 108 species of carnivores have been exploited for their body parts for use in TM worldwide. The unsustainable use of plants and animals in TM is a threat, directly and indirectly, to the conservation of many rare and endangered species, and it has caused declines in wild populations due to overexploitation [6, 23–25]. Nearly half of species listed as Near Threatened to Critically Endangered in the International Union for the Conservation of Nature (IUCN) Red List of Threatened Species are used as medicine or food [26]. Cambodia is considered rich in biodiversity; however, many species are listed as threatened in the IUCN Red List [27, 28]. In Cambodia, all wildlife species are considered to be state property, and hunting rare and endangered species is illegal [29]. However, many large mammal species have become nationally extinct such as Javan Rhino (*Rhinoceros sondaicus*), Kouprey (*Bos sauveli*), and Indochinese tiger (*Panthera tigris tigris*), while other large mammal populations continue to decline [28, 30, 31]. While domestic trade in wildlife meat and TM is considered to be one of the main threats to wildlife, Cambodia also serves as a source and transit point for international illegal wildlife trade [9, 28, 32]. Growing demand for wildlife products increases hunting pressure; between 2010 and 2019 more than 234,000 illegal snare traps were removed from five protected areas in Cambodia, and this threat is ongoing [33].

In this context, we conducted semi-structured interviews with TKM practitioners in rural areas of Cambodia where people have limited access to Western medicine and are highly reliant on TM, as well as teachers of TKM at the National Center of Traditional Medicine (NCTM). This study has objectives to explore in detail: (1) how TKM is practiced in modern times; (2) how TKM practices are transmitted and how they have adapted; (3) and the role of wildlife part remedies in TKM. The insights gained will inform conservation initiatives and management plans to reduce demand for products from threatened wildlife, and identify ways to promote sustainable behaviours. Throughout this article TM and TKM refer to both plant- and animal-based medicines, unless otherwise specified.

## Methods

This study was carried out between June 2018 and January 2019. We used a semi-structured interview (SSI) to discuss Traditional Khmer Medicine (TKM) and the use of wildlife. The interview instrument was designed based on a SSI guide tested in Cambodia in September 2016 [34] and was refined to align with this project’s aims through collaborative input from all authors. The interview instrument (Additional file 1: Appendix I) was prepared in English and translated to Khmer by a professional translation service and crosschecked by the lead author, who speaks both languages. In the English translation of the interview guide, biomedicine was referred to as “Western medicine”, which also aligns with how biomedicine is translated in Khmer, as *barang* (foreign) medicine or *peyt (doctor)* medicine. In this manuscript, we have chosen to refer to it as “biomedicine” according to recent literature around this issue (e.g. [35]).

The interviewed TKM practitioners were from a variety of professional levels including traditional Khmer healers (*Kru Khmer*), traditional birth attendant (TBA)/grandmother midwives (*Chhmob boran* or *Yeiy mop*), and Buddhist monks—who practice TKM together with performing exorcism ceremonies and spiritual healing based on Buddhist principles [36]. Convenience sampling was conducted due to the unofficial nature of information about TKM practitioners in the study area. Upon arrival at the study sites, we asked for information about TKM practitioners’ location from village chiefs. With so few TKM practitioners present in each village, we attempted snowball sampling in order to seek out potential interviewees, but found that TKM practitioners did not like to disclose the details of other practitioners because they were direct competitors for clients.

Additionally, using the SSI guide, key informant interviews were conducted with TM healers/teachers and researchers from the National Center of Traditional Medicine (NCTM), Cambodian Traditional Healer Association (CaTHA), and Association of Traditional Cambodian Medicine (ATCM) located in Phnom Penh (the capital city of Cambodia), in order to help obtain more understanding around the topic of wildlife use in TKM. Three of the key informants were also practitioners and are included with the other TKM practitioners in analysis, while two are not. Instead, we cite their insights and knowledge separately within this article. These key informants were given anonymous numeric identifiers for use in this article, e.g. “Key Informant #1”.

This study was approved by provincial authorities of both provinces in which interviews were conducted and the Cambodian Ministry of Environment. Ethical approval was granted by the Miami University of Ohio’s Internal Review Board (Protocol ID: 02106e). All respondents were given random IDs, assured of their anonymity, and informed that they could end the interview at any point. All interviews were conducted in Khmer, the main language in Cambodia, by the lead author. The lead author was assisted by a Khmer notetaker who directly transcribed the interview as it happened. To further ensure the safety of the respondents, the physical interview records were kept in a sealed cabinet within a locked office in Phnom Penh that only the author team had access to. The recorded data were translated from Khmer to English by a professional translation service. Analysis of the data was then performed with NVivo (version 12) for visualizing, classifying, sorting, and arranging the data into themes.

## Study area

**Fig. 1:**
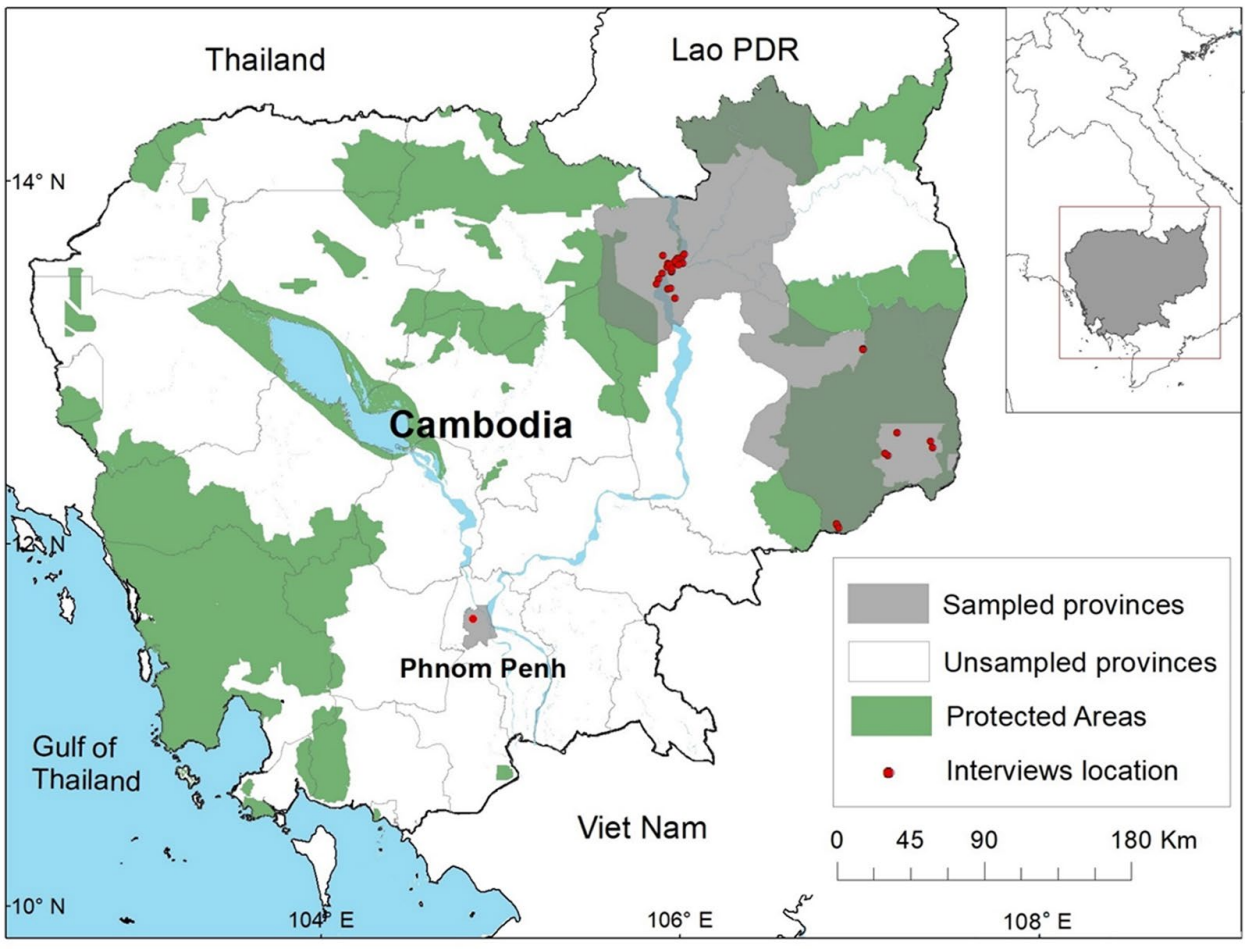
Map of Cambodia showing the location of the study areas (grey) and interview locations (red dots) in relation to Protected Areas (green)

Traditional Khmer Medicine (TKM) practitioners were interviewed in rural villages in Mondulkiri Province (12° 27′ N 107° 14′ E) and Stung Treng Province (13° 31′ N 105° 57′ E), in East and Northeast Cambodia (Fig. 1). With a total area of 11,092 km^2^, Stung Treng is the least densely populated province in Cambodia [37] and is known as the Cambodian “Upper Mekong” province, adjacent to the international border of the Laos (Fig. 1). In Stung Treng, the most prevalent ethnicity (other than Khmer) is Lao [38]. Mondulkiri has a total area 14,288 km^2^, is one of the largest provinces in Cambodia, and borders Vietnam (Fig. 1). Mondulkiri has a diversity of Indigenous groups living within the province, with the most common Indigenous group being the *Bunong* people [39]. Both provinces are known to be less developed and were selected as study areas based on their characteristics of having rich biodiversity and large intact forest; in addition, human population density is relatively low and there are fewer medical clinics where people have access to biomedicine [40]. Thus, we predicted that in these areas people may be more reliant on traditional medicine.

The National Center of Traditional Medicine (NCTM), Cambodian Traditional Healer Association (CaTHA), and Association of Traditional Cambodian Medicine (ATCM) are located in Phnom Penh—the capital city of Cambodia with a population of 2.3 million, including a large proportion of rural–urban migrants [41].

## Results and discussion

### Demographic information

We interviewed a total of 35 people comprised of TKM practitioners in Stung Treng (n = 20) and Mondulkiri (n = 10), and key informants (n = 5) from NCTM, CaTHa, and ATCM in Phnom Penh City. The key informants were TM teachers and researchers who also actively practice TKM; therefore, their responses are pooled with other TKM practitioners. Overall, we interviewed 23 men and 10 women, with an average age of 62 years. All of the participants still practice TM with an average of 25 years’ experience, with only 12% (4/33) of interviewees having practiced for less than 5 years. The respondents saw an average of 31 people/month who came to get treatment or purchase TM. The interviewees’ ethnicities were: Khmer (20/33, 60.6%), Khmer-Lao (8/33, 24.3%), Lao (3/33, 9.1%), Bunong (1/33, 3%), and Khmer-Chinese (1/33, 3%).

### TKM training

The total number of TM practitioners in Cambodia is not known because there is no official registry. However, it has been documented that there is at least one in every village [42]. These practitioners see their patients and teach the next generation, either at their home or a Buddhist temple [42]. Generally, TKM practitioners are well-respected people in the villages, or are a respected Buddhist monk [16]. In our study, 97% (32/33) of the respondents (including the key informants in Phnom Penh City) began practicing TM without enrolling in any academic training, with only one respondent (3%) reporting having been enrolled in a related training course in neighbouring Vietnam. The respondents mostly learned from their kin or close relatives who were TKM practitioners (48.5%, 16/33) by starting to follow those people to collect the medicine ingredients in forests and start to remember the remedies without a written record or reference book. Some of them learned from monks or other peer TKM practitioners (33%, 10/33) and learned by exploring themselves through the personal books or remedies that they knew or heard of from elder people in their villages (24.2%, 8/33). About 66.6% (20/33) of TKM practitioners said they do not teach the practice to their next generation or children, claiming that the young people have less interest or no talent in remembering the medicines. In the case of spiritual healers (*boramey*), it is believed that the healer is embodied by spirits that provide the ability to heal, and therefore not everyone has the ability to learn this TKM practice [43]. Respondents also noted, as reasons for not teaching TKM, the availability of biomedicine, the low earnings from being a TKM practitioner, and the difficulty of finding the medicine ingredients from the forest.

“I learnt it from my father, when he taught me about the plants that could be used as herbal medicine. I learnt at Kandal Province since I was around 20 to 26 years old, but I hadn’t collected the herbal medicine for selling until I was 30 to 36 years old. [Then] I started making traditional medicine, until today [approximately twenty years]. I have never taught it to my children. Even though they know the plants, they don’t know how to use it, and they don’t have talent to do it.” [M, 56, Khmer, TKM practitioner, Mondulkiri]

“I learnt in the forest by myself [learnt from Neak Sachang - hermit monk, referring to the spirit that lives in the forest or cave]. I learnt since I was 8 years old as I was lost in the forest for the whole week. After that I moved to live in the cave behind my house, but I left it later after the workers at that mountain didn’t allow me to live there… I moved here in 1998. When I cured them; for example, the mother is sick, and her child comes to ask me for help, I will collect the information and tell to my peer who lives in the forest [Neak Sachang - hermit monk spirit]. They will tell me whether the disease can be cured or not.” [M, 37, Khmer, TKM practitioner and Khmer healer, Stung Treng]

“Before I become a Khmer healer and TKM practitioner, I was a nurse who studied about TKM since 1949… During Pol Pot Regime, in 1970, I was assigned to control the usage of medicine…8 years after Pol Pot Regime, I changed to work in the army and became a captain… After that, I went to study about TM that could be made as pills or injections in Danang, Vietnam. I also studied it from Chinese guys who came to teach in Cambodia… I’ve never taught my kids how to make the traditional medicine, but I’ve done it with modern medication. I am retired now [from the army], so I will continue doing my job as Khmer healer (Kru sdos plom, Kru snea) and traditional Khmer medicine practitioner.” [M, 84, Khmer, TKM practitioner and Khmer healer, Mondulkiri]

Unlike TMs practicing in some countries in Asia (e.g. China, India, Japan, South Korea, Vietnam), TKM is generally practiced by the private sector; mostly informal practitioners who live in rural or remote areas [13]. TKM has not yet been integrated with biomedicine, or included into the National Health Strategic plan and health insurance [42]. None of the informants that we interviewed at study sites in the provinces had the formal license to practice TM, only the informants in Phnom Penh had official licenses to practice TM. In 2010, the first *Traditional Medicine Policy of Kingdom of Cambodia* was adopted, which states that TM shall be an important component of the healthcare system in Cambodia, with the goal of helping to maintain and improve the healthcare system in remote and poor areas of Cambodia [13]. The National Center of Traditional Medicine (NCTM) was established that year as the implementing agency with the support of the Ministry of Health and international partners. This centre strives to improve the quality of TM and products in Cambodia, advocates for the inclusion of TKM in the nation’s primary health care, and promotes the integration of TMs as biomedicine through scientific research, and regulation of production. According to Key Informant #1, the NCTM provides capacity building to TM practitioners by running a 6-month training course (also supported by [9, 13]). The course is more focused on plant-based TM and encourages the use of home-grown plants. Key Informant #1 further stated that after this course TM practitioners are awarded the certificate that enables them to apply to the Municipal or Provincial Health Department for a business license as formal TMs practitioner and open their shops (also supported by [42]). This training course was available for all types of TM practitioners free of charge, including the living expense during training at the centre up until 2013. However, as Key Informant #1 noted, after 2013 the centre had no funding to support trainees so they had few participants. Most TKM practitioners live in the rural area and are poor. It is difficult for them to travel to the city, and/or they are old and cannot read or write (also T. Lim, per. obs.). Therefore, TKM training and practice remains largely informal and unregistered (T. Lim, per. obs.).

### Roles of TKM practitioners

All respondents who live in rural or remote areas commonly practice in their private home without a formal license. Their roles commonly involve preparing (90.9%, 30/33) and selling medicine (90.9%, 30/33), more so than applying direct treatment (39.4%, 13/33). Respondents mostly collect medicinal ingredients themselves (75.8%, 25/33) and provide general consulting or advising regarding medicine use (69.7%, 23/33). The interviewed practitioners can expand their catchment areas by becoming well known and offering a wide range of medicines. The interviewed practitioners reported never having used media advertising; rather they become known through word-of-mouth, or through recommendations from patients who have experienced getting better or cured by their treatment. The practitioners typically become well known for their ability to treat one or two specific illnesses. Respondents reported that it is getting easier to interact with patients from distant areas or other provinces due to the possibility to conduct consultations over the phone and the ease at which medicine can be sent by taxi to costumers/patients. TKM practitioners in rural areas also provide advice to patients on medicine or self-treatment, for which the patient can volunteer to pay. The patients can go to buy the medicines themselves from markets or go looking for ingredients from the forest.

### TKM interaction with biomedicine

TKM practitioners only practice their TM remedies in the private sector (i.e. they are not state-funded), following what they learn from their peers, without the use of written text [9, 13]. Of the respondents, 67% (22/33) claimed that the number of people getting TM from them has increased compared to when they started, despite the increasing accessibility of biomedicine during the more than 40 years since the Khmer Rouge regime—a significant memory anchor for people in Cambodia. The majority of respondents believed that it is more effective to get treatments from both biomedicine and TKM at the same time without the risk of harmful reactions or side effects. Respondents said that TKM can be used as alternative when biomedicine is not effective enough or is unaffordable. This may contribute to the practice in Cambodia of using the treatments together. Some respondents claimed that there are types of illnesses that are best treated using TKM and for which biomedicine or doctor cannot cure. Those include the illnesses that people believe are caused by spiritual reasons “*Trov Ampeur*”, referring to the illness that is being overpowered by ghost spirits or cursed by “dark power” people who hate them. For these ailments, TKM practitioners must combine medicinal and spiritual techniques. TKM is also considered to be best for common illnesses such as women-related illnesses and maternal care (e.g. vaginal discharge, illnesses after giving child birth “*Toas*”), stomach problem, loss of appetite, measles, broken bones, and body pain [13,34, T.Lim, per. obs.]. TKM is also used for daily consumption to prevent the illnesses, e.g. for general healthcare people use herbal medicine boiled with daily drinking water, and porcupine (*Hystricidae* spp.) stomach wine or bear bile wine for daily drink during the post-partum period [35].

In contrast to the 67% of respondents who believed people coming to them had increased, 33% (11/33) of the respondents claimed that they had decreased in popularity because of the increased accessibility of biomedicine. One respondent mentioned that the increasing price of TM treatment is one reason people choose biomedicine. Respondents also reported difficulty in finding the medicine ingredients due to the loss of forest and increased agriculture. One practitioner stated that he is no longer able to sell medicine to tourists (Khmer travelling to visit the forest from the city) where he used to, because the place was recently converted to a private tourism resort. According to the key informants in Phnom Penh, TKM was perceived to have been greatly used in the country from the late 70’s to late 80’s during and directly after the Khmer Rouge regime when there was no importation in biomedicine or other TMs into Cambodia. According to Key Informant #2, in the 1990s after the political change resulting from the coming of the United Nations Transitional Authority in Cambodia (UNTAC), Cambodia regained access to biomedicine, which has now taken over as the first choice of healthcare. According to Key Informants #2 and #3, even though wide accessibility of biomedicine has discouraged the use of TM, it can still be used for primary healthcare in this country, and it is especially popular among villagers who live in rural or remote areas where there is limited Western healthcare (also supported by [9]). TKM was previously adopted into the country’s healthcare policy, but there was little effort to promote it. Furthermore, according to Key Informant #2, the difficult process of formally registering as a TKM practitioner has meant that TKM is less popular than biomedicine.

### Use of wildlife in TKM

**Fig. 2:**
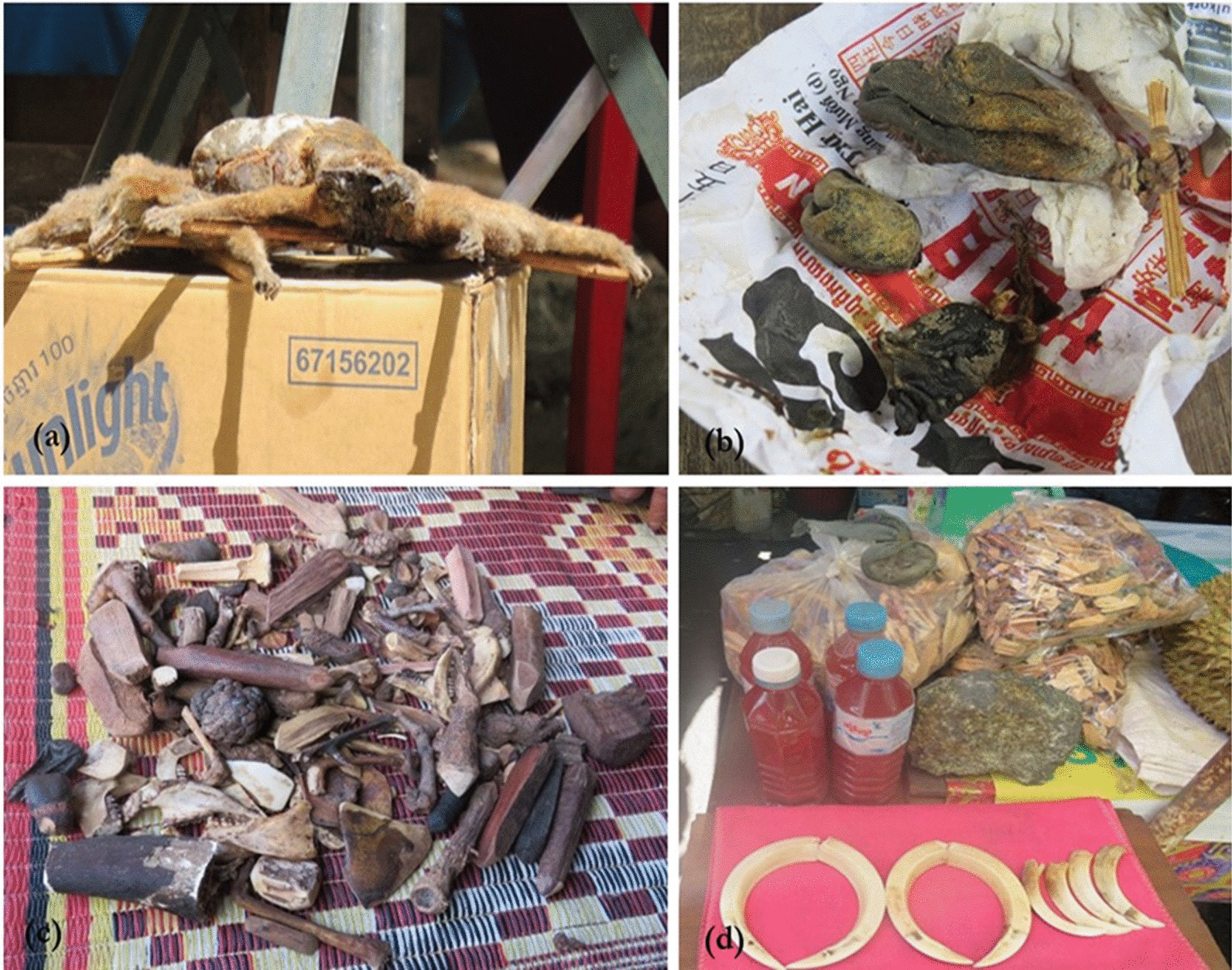
Wildlife parts were used for Traditional Khmer Medicine (TKM) during survey **a** dry loris before using as medicine, **b** dry wild boar gall bladder, **c** various animal parts include wildlife parts and plants use as “Rubbing medicine”, **d** porcupine blood wine and others wildlife products sold by practitioners at tourist site. Credit: *Lim Thona*

According to Key Informants #4 and #5, TKM uses plants, animal parts (both of wildlife and domestic animals), and minerals, although there is greater emphasis on using plants (also [9, 18]). The preparation of the animal-based TM can consist of parts of several animals combined or just a single animal part. Animal parts can be used in TKM by rubbing with water or coconut water (“Rubbed medicine”; in Khmer “*Thnam Dos*”) or are soaked with alcohol for using, while also burning and grinding (Fig. 2). This knowledge was clearly emphasized by all respondents in this study, and wildlife species that have been used were also cited in the Khmer medicine pharmacopeia. This book is used in the National Center for Traditional Medicine to teach foundation year of health subject students and local TM practitioners about the animal-based medicine [18]. Those animals highlighted include mammals, reptiles [44], birds, aquatic animals, and insects, along with the specific organs to be used, e.g. rhino horn, tiger bone, bear gall bladder, porcupine stomach, or pangolin scale [18]. Key Informant #1, interviewed at the National Center for Traditional Medicine, stressed that the reference to the use of wildlife in TKM was simply an introduction to the history of TKM that also includes wildlife parts; however, they do not practice or teach in detail about the use of wildlife in treatments for aliments because the centre was guided by the Ministry of Environment regarding protected species. Most of the high-profile species used in TM are protected in Cambodia and asking about the wildlife medicine in our survey was challenging—most TKM practitioners and key informants interviewed were hesitant to speak much with us due to the apparent sensitivity of this topic.

Nonetheless, in this study, respondents reported having used mammal species (54%) in TKM more than other taxa, followed by reptiles (29%), and birds (18%). More than half of the practitioners interviewed (58%, n = 19) reported that they have been using the same wildlife species since they started practicing (before 2013) and in past 5 years (2013 to time of survey), but relatively little animal-based medicines are used compared to plant-based medicines (Table 1).

**Table 1:** Wildlife species reported in this study as being used in TKM by practitioners (n = 33)

Common name	Species present in Cambodia	Authority **n**ame*	IUCN Red List Status**	Cites appendix***	Practicing (before 5 years ago)		Practicing (during the past 5 years)		Parts used
					n	%	n	%	
Loris	*Nycticebus bengalensis* *Nycticebus pygmaeus*	Lacépède, 1800 Bonhote, 1907	EN EN	I	14	42.4	16	48.5	Whole body, stomach
Mainland serow	*Capricornis sumatraensis*	Bechstein, 1799	VU	I	10	30.3	13	39.4	Bone, horn, blood and skin
Porcupine	*Hystrix brachyura* *Atherurus macrourus*	Linnaeus, 1758 Linnaeus, 1758	LC LC	–	10	30.3	9	27.3	Stomach, canine teeth, and blood
Tiger	*Panthera tigris*	Linnaeus, 1758	EN	I	5	15.2	5	15.2	Bone, gall bladder, canine teeth and claw
Bears	*Ursus thibetanus* *Helarctos malayanus*	Cuvier, 1823 Raffles, 1821	VU VU	I	12	36.4	5	15.2	Gall bladder/bile, claw, blood, paw, teeth and skin
Asian elephant	*Elephas maximus*	Linnaeus, 1758	EN	I	6	18.2	4	12.1	Molar, bone, ivory penis and skin
Wild boar	*Sus scrofa*	Linnaeus, 1758	LC	–	5	15.2	4	12.1	Fat
Deer	Cervidae	–	EN VU LC	–	4	12.1	4	12.1	Horn, bone, penis, leg and hoof
Sunda pangolin	*Manis javanica*	Desmarest, 1822	CR	I	2	6.1	2	6.1	Whole body, scale, and blood
Gaur	*Bos gaurus*	C.H. Smith, 1827	VU	I	2	6.1	1	3.0	Horn and bone
Greater hog badger	*Arctonyx collaris*	F.G. Cuvier, 1825	VU	–	3	9.1	1	3.0	Fat, bone, teeth and gall bladder
Irrawaddy dolphin	*Orcaella brevirostris*	Owen in Gray, 1866	EN	I	2	6.1	1	3.0	Bone
Langur	*Trachypithecus* sp. *Pygathrix* sp.	–	–	–	2	6.1	1	3.0	-
Others: Dhole, Big cat sp.		–	–	–	1	3.0	1	3.0	-
Asian openbill	*Anastomus oscitans*	Boddaert, 1783	LC	–	2	6.1	2	6.1	Beak
Hornbill sp.	*Buceros* sp.	–			2	6.1	2	6.1	Beak
Lesser coucal	*Centropus bengalensis*	Gmelin, 1788	LC	–	1	3	1	3	Whole body
Woodpecker	**–**	**–**	–	–	1	3	1	3	Whole body
Tortoise sp./Softshell turtle	–	–	–	–	7	21.2	3	9.0	Whole body, blood, head and chest
Python	*Python* sp.	*–*	–	–	4	12.1	2	6.1	Gall bladder
Cobra	**–**	**–**	–	–	1	3	1	3	Whole body, blood and gallbladder
Snake	**–**	**–**	–	–	2	6.1	1	3	Whole body and blood
Crocodile	**–**	**–**	–	–	2	6.1	1	3	Teeth
Toad	**–**	**–**	–	–	2	6.1	2	6.1	Whole body

Others: snail sp. (marine and freshwater), Leech, Ka chang (local name of small bird)

^*^https://www.iucnredlist.org/

^**^https://www.iucnredlist.org/; LC, least concern; VU, vulnerable; EN, endangered; CR, critically endangered

^*^**https://www.speciesplus.net/

Among the reported animals used (Table 1), 6 species are classified as Least Concern, 6 species as Vulnerable, 6 species as Endangered, and 1 species as Critically Endangered [45] by the IUCN Red List of Threatened Species [46]. Of those, 10 species are included in the CITES Appendices I, which prohibits international commercial trade. These reported species have been confirmed a decade ago as being found in the illegal wildlife trade and used as traditional medicine in the country [9, 28, 47]. The species of some wildlife used cannot be classified here due to lack of specificity by interviewees. However, the findings of this study highlight the ongoing nature of this threat to protected species.

When asked which animal parts are most commonly used in respondent’s practices, lorises (*Nycticebus* sp.) were the first mentioned and most frequently used by respondents in the 5 years prior to the survey, and before (49% of respondents, n = 16). Lorises were claimed to have a healing agent for post-partum disorders such as *Toas* (one respondent pointed to specifics such as *Toas sawsaye*—relapse from back to work too soon; and *Toas chimney*—relapse from eating the wrong foods); and *Sawsaye kchey—immature blood vessels* [48, 49], and was stated to be commonly used by women for this purpose, as well as both genders for various ailments such as wounds and dermatosis, body pain, broken bones, internal bruising, and gastroenteritis. Several studies have shown that lorises are not just the most used taxa in TKM, but also by Chinese, Vietnamese and even Indigenous Traditional Bunong medicine, and is one of the most widely traded wildlife taxa in Cambodia [9, 47, 50, 51].

In our study, Mainland serow (*Capricornis sumatraensis*) (39%, 13/33) was the second most commonly used taxa—those parts include bone, horn, blood and skin. TKM practitioners prescribe them for broken bone, gynecological/uterine ailments, healing wounds, and measles. Serow has also been found to be used frequently and for similar ailments, in a study conducted in northern Laos [52]. Porcupine (27%, 9/33) was the third most commonly used taxa as reported by respondents. In particular, especially popular was porcupine stomach that was mainly used for women with post-partum disorders, and body pain for all genders. Porcupine stomach is also known to be used in Vietnam, for TM purposes [53].

Lorises and porcupine were also reported as the two most requested animal parts by consumers and patients when they visited TKM practitioners. The informants claimed that these animals’ parts are easy to find and buy from local markets and even by requesting hunters target these species. One respondent in Stung Treng Province claimed that he can buy one loris for 20,000 KHR (5 USD) and one whole porcupine’s stomach for 40,000 KHR (10 USD). This price is consistent with that of studies conducted over a decade ago by [9] and [50]. Post-partum disorders are known to be commonly treated with TKM using these species [50, 54], and this knowledge is well known among general villagers without prescription or consulting with practitioners [9, T. Lim, pers. comm., 2018].

Tiger (*Panthera tigris*) parts were the fourth most commonly reported (15.2%, 5/33) parts prescribed in past 5 years. There was little detailed information from respondents regarding tiger part use, although the parts used were reported as being bone, gallbladder, canine teeth, and claws. Tiger bones are rubbed together with other wildlife parts and medicinal plants as “Rubbing medicine”, while canine teeth and claws were reportedly used as necklace pendants to bestow power and offer protection to the wearer. According to [30], the tiger population is possibly extinct from the Cambodian forest, with the last image captured by camera trap in 2005. The decline of the population was driven by over hunting and linked to the regional wildlife market during continued armed conflict between 1953 and 2005 [55]. Thus, it is possible that the tiger parts used by our respondents may have been sourced from farms or the wild in neighbouring countries, or may have been fakes (e.g. cow bone, bear canines). Bear parts (Asiatic black bear *Ursus thibetanus*, Sun bear *Helarctos malayanus*) are the fifth most commonly prescribed wildlife parts reported by respondents in the 5 years prior to the survey (15.2%, 5/33), yet were cited as being the most commonly prescribed parts before that (36.4%, 12/33). The parts used include gallbladder/bile, claws, blood, paw, teeth and skin. Bear gallbladder was reported as the most sought-after and most valued medicine among the others parts.

### Sourcing wildlife parts

As shown in Table 1, the supply of wildlife ingredients has dropped in the 5 years prior to the survey. All TKM practitioners who reported using wildlife noted that, nowadays, wildlife ingredients in TKM are more difficult to find and more expensive than before. Moreover, it is against the law to buy and/or use these parts. To obtain them, respondents reported that they need to source wildlife discreetly from trusted traders. Practitioners that use wildlife in medicine (58%, n = 19) reported that prior to 5 years ago, they commonly obtained those wildlife parts directly from hunters (63%, 12/19). Apart from that, these respondents reported sourcing wildlife by personally hunting in forest (16%, 3/19), buying from middlemen (16%, 3/19), and getting from family or relative (without clear sources) (16%, 3/19). One practitioner in Stung Treng reported obtaining wildlife/wildlife parts from a relative who lives in Laos, which borders the province. During the past 5 years, however, practices have shifted. While practitioners continue to obtain wild animal parts through direct contact to hunters, it is less common than before (37%, 7/19). Instead, they have also​ started obtaining wildlife by buying from markets in the provincial capital towns or Phnom Penh Capital City (32%, 6/19), by continuing to use what they had from previous years (16%, 3/19), getting from family or relative, as before, (16%, 3/19), getting the supply from Lao PDR (11%, 2/19), self-hunting in forest (5%, 1/19), buying from middleman (5%, 1/19), and buying from TCM shops in Phnom Penh (5%, 1/19). This latter strategy for acquiring wildlife products, while only utilized by one individual in our sample, indicates similarity in the medicinal strategy of TKM and neighbouring TM practices.

Respondents reported that wildlife used in their practice was sourced from the wild, as opposed to from a commercial wildlife farm, both in past 5 years and before. Furthermore, in last 5 years when wildlife parts were reported to become harder to find, they started looking to purchase from external markets, including from Phnom Penh and others country like Laos. During this study, wildlife parts (dried or steeped in alcohol), live wild animals and wildlife meat were observed openly for sale in the Stung Treng town market, while in Mondulkiri a practitioner was observed selling TM and wildlife parts at a local tourist attraction, for the benefit of people visiting from Phnom Penh and others provinces (Lim, T. pers. obs. 2018).

## Conclusion

This study has contributed to understanding about the practice of Traditional Khmer Medicine (TKM) in modern-day Cambodia, and in particular the role it plays in the use of wildlife. TKM practitioners still play important roles in people’s health care in Cambodia. TKM is still chosen as a treatment combined with biomedicine, or may still be the only option in rural areas in Cambodia despite increased accessibility of biomedicine after the Khmer Rouge regime. TKM treatments were also chosen when biomedicine or doctor cannot cure, e.g. illnesses that require spiritual healing. The rural TKM practitioner commonly acts in roles of collecting, preparing, selling, and advising about medicine to their patients to treat themselves at home, rather than the TKM practitioner providing treatment. TKM practitioners generally practice in the private sector and informally, and practice what they have learnt from their peers without consulting a standard written text and/or enrolling in academic training. This knowledge has been transferred the same way from generation to generation. The National Center of Traditional Medicine (NCTM), based in the capital city Phnom Penh, is the only national academic training available to provide capacity building to local TM practitioners to become licensed practitioners.

Our study highlights the ongoing threat to protected species, with more than 50% of interviewed rural TKM practitioners still prescribing and using wildlife medicine. Reported wildlife species include endangered and threatened carnivores, including tigers and bear species. All wildlife were explained by respondents to have a long use in TKM history, and reported wildlife species were found in the pharmacopoeia book used by the National Center for Traditional Medicine, which is used for teaching and introducing wildlife medicine to medical students and TM practitioner trainees. 28 identified wildlife species have been used, which include CITES Additional file 1: Appendix I species and species listed as Endangered and Critically Endangered on the International Union for the Conservation of Nature Red List of Threatened Species (Table 1). Wildlife parts were all reported to be from practitioner’s direct contact to hunter or finding in local markets, i.e. products come directly from the wild. Species like lorises and porcupine were most highly demanded and used in TKM. They are primarily prescribed for women’s post-partum illnesses (*Toas and Sawsaye kchey*). This behaviour raises concern for future species conservation as well as for women’s health in Cambodia, both key issues in the country and contributors to Cambodia’s failure to achieve global targets such as the United Nations Sustainable Development Goals (e.g. [56, 57]). Addressing both of these issues can be accomplished through implementing interventions that promote biomedicine as a more effective treatment for women’s health concerns in rural Cambodia [35].

TKM practicing is still important for local primary healthcare in rural areas and we found that consumers trust the knowledge and follow the suggestions of TKM practitioners. In those areas of Cambodia that continue to be more rural and isolated from biomedical facilities, behaviour change interventions should be implemented that encourage use of plants over animal species, when seeking TKM treatment options. A possible leverage opportunity could be to strengthen pride in natural heritage, following the framework of successful Rare Pride campaigns [58]. These campaigns could recruit TKM practitioners as spokespeople, drawing on their reliance on the forest and the wildlife within to encourage conservation ethos. However, such campaigns would need to be carefully researched, designed, and tested to ensure that they do not cause unsustainable exploitation of plant species, and/or engender greater interest in animal-based TKM use.

## Supplementary Information

**Additional file 1: Appendix I**. Interview guide: Traditional Khmer Medicine practitioners.

## Data Availability

The datasets used and/or analysed during the current study are available from the corresponding author on reasonable request.

## Abbreviations

ATCM: Association of Traditional Cambodian Medicine
CaTHA: Cambodian Traditional Healer Association
CITES: Convention on International Trade in Endangered Species of Wild Fauna and Flora
CR: Critically Endangered
EN: Endangered
IUCN: International Union for Conservation of Nature
LC: Least Concern
NCTM: National Center of Traditional Medicine
TBA: Traditional Birth Attendant
TM: Traditional medicine
TCM: Traditional Chinese Medicine
TKM: Traditional Khmer Medicine
UNTAC: United Nations Transitional Authority
VU: Vulnerable
