# Supplementary material for: Traditional Khmer Medicine and its role in wildlife use in modern-day Cambodia
Source: J Ethnobiol Ethnomed. 2022 Sep 24;18:61. doi: 10.1186/s13002-022-00553-5 (PMC9508725; doi:10.1186/s13002-022-00553-5)
Supplement: Supplementary file 1 — Additional file 1: Appendix I. Interview guide: Traditional Khmer Medicine practitioners. [file 13002_2022_553_MOESM1_ESM.pdf]

Interview Guide: Traditional Khmer Medicine practitioners

Interview Number:

Date:

Village:

Commune:

Province:

Interviewer:

Note Taker:

Other People Present:

How did you meet the interviewee? (Met Randomly, Introduced as TKM Practitioner, Introduced as someone who knows about wildlife/bears):

Consent to be interviewed? (Yes/No):

Reason given for not consenting/stopping the interview:

I. Demographic information

1. Age
2. Gender
3. Ethnicities
4. What kind of the Traditional Khmer Practitioner are you?
5. Do you still practice this work? (if NO, when did you stop?)
6. How long do you practice this work?
7. How many people do you treat in one month?

I. Understand the roles of TKM practicing

8. How and where did you learn about how to practice TKM? Who taught you about this? And how did you transfer this practicing to your next generation?

9. What roles do you commonly play being involved TKM practicing (applying treatment, preparing medicine, selling TKM medicine, or guiding/ advising)?
10. Can you tell me, how the TKM practice has changed over the past 40 years (since from the Khmer Rouge)? *(Here, the question can be revised for asking base on the years of the practitioner's experiences or from what they have known from their teachers)*
11. Over the past 40 years (since the KR Time) how has the TKM been influenced by other TMs e.g. Chinese, Vietnamese, Lao, Cham?
12. Over the past 40 years (since the KR Time) how has the TKM been influenced by Western medicine?

II. Understand about the history of the wildlife uses in TKM

13. Based on your knowledge about the TKM, what kinds of animals are/used to be used in TKM?

    If there're no animals practicing in the TKM, is it different TM that does animal base?

Asking about the present practicing: In past 5 years until now (previous national election 2013 to now)

14. Can you tell me about what are the animals are using for your medicine practicing in past 5 years (2013) until now?
15. Among them what are the top 10 animals parts most commonly prescribed animals?
16. What is the most commonly requesting from your patients/the people that come to see you?

Asking about the past practicing (follow Q14-16): how about before last 5 years (since practicing to 2013)?

17. Can you tell me about what are the animals are using for your medicine practicing since you started practicing until 2013 (before the last 5 years)?
18. Among them what were the top 10 animals parts most commonly prescribed animals?

19. What was the most commonly requesting from your patients/the people that come to see you?

III. *Understand about the history of the bear part uses in TKM*

20. What kinds of the bear parts can be in TKM practice? What ailments are bear parts used for?

Part	Ailment	Alternatives (Qt 24)
Bile:		
Gallbladder:		
Blood:		
Claws:		
Teeth:		
Paw:		
Bone:		
Other:		

21. What bear parts have you used in your practice?
22. How has the use of bear parts in TKM changed in the past five years? And the past 40 years?
23. Are you more likely to [a] supply bear products if someone requests them or [b] suggest bear products to people suffering from an ailment that you know can be treated with bear products [choose only one option]

IV. **Understanding adaptation to change**

24. Over the past 40 years (since the KR Time) how has the supply of wild animal ingredients for TKM changed? Easier/more difficult to find? Where did ingredients

come from before/where now? Cheaper/more expensive? What about for bear parts in particular?

25. If it is more difficult or more expensive to find wild animal ingredients now, what do you use/prescribe instead to treat the ailment?
